# Functional optical coherence tomography at altitude: retinal microvascular perfusion and retinal thickness at 3,800 meters

**DOI:** 10.1152/japplphysiol.00132.2022

**Published:** 2022-06-30

**Authors:** Jacquie Baker, Mohammad A. Safarzadeh, Anthony V. Incognito, Nicholas G. Jendzjowsky, Glen E. Foster, Jordan D. Bird, Satish R. Raj, Trevor A. Day, Caroline A. Rickards, Natalia Zubieta-DeUrioste, Usman Alim, Richard J. A. Wilson

**Affiliations:** ^1^Department of Cardiac Sciences, Libin Cardiovascular Institute, Cumming School of Medicine, University of Calgary, Calgary, Alberta, Canada; ^2^Department of Physiology and Pharmacology, Cumming School of Medicine, University of Calgary, Calgary, Alberta, Canada; ^3^Hotchkiss Brain Institute, Cumming School of Medicine, University of Calgary, Calgary, Alberta, Canada; ^4^Alberta Children’s Hospital Research Institute, Cumming School of Medicine, University of Calgary, Calgary, Alberta, Canada; ^5^Division of Respiratory and Critical Care Physiology and Medicine, The Lundquist Institute for Biomedical Innovation at Harbor-UCLA Medical Center, Torrance, California; ^6^Centre for Heart, Lung, and Vascular Health, School of Health and Exercise Sciences, University of British Columbia, Kelowna, British Columbia, Canada; ^7^Department of Biology, Faculty of Science and Technology, Mount Royal University, Calgary, Alberta, Canada; ^8^Department of Physiology & Anatomy, University of North Texas Health Science Center, Fort Worth, Texas; ^9^High Altitude Pulmonary and Pathology Institute (HAPPI – IPPA), La Paz, Bolivia; ^10^Department of Computer Science, University of Calgary, Calgary, Alberta, Canada

**Keywords:** cerebral microvasculature, functional optical coherence tomography, high altitude, hypoxia, retinal microvasculature

## Abstract

Cerebral hypoxia is a serious consequence of several cardiorespiratory illnesses. Measuring the retinal microvasculature at high altitude provides a surrogate for cerebral microvasculature, offering potential insight into cerebral hypoxia in critical illness. In addition, although sex-specific differences in cardiovascular diseases are strongly supported, few have focused on differences in ocular blood flow. We evaluated the retinal microvasculature in males (*n* = 11) and females (*n* = 7) using functional optical coherence tomography at baseline (1,130 m) (*day 0*), following rapid ascent (*day 2*), and prolonged exposure (*day 9*) to high altitude (3,800 m). Retinal vascular perfusion density (rVPD; an index of total blood supply), retinal thickness (RT; reflecting vascular and neural tissue volume), and arterial blood were acquired. As a group, rVPD increased on *day 2* versus *day 0* (*P* < 0.001) and was inversely related to $Pa_{O}{}_{{}_{2}}$ (*R^2^* = 0.45; *P* = 0.006). By *day 9*, rVPD recovered to baseline but was significantly lower in males than in females (*P* = 0.007). RT was not different on *day 2* versus *day 0* (*P* > 0.99) but was reduced by *day 9* relative to *day 0* and *day 2* (*P* < 0.001). RT changes relative to *day 0* were inversely related to changes in $Pa_{O}{}_{{}_{2}}$ on *day 2* (*R^2^* = 0.6; *P* = 0.001) and *day 9* (*R^2^* = 0.4; *P* = 0.02). RT did not differ between sexes. These data suggest differential time course and regulation of the retina during rapid ascent and prolonged exposure to high altitude and are the first to demonstrate sex-specific differences in rVPD at high altitude. The ability to assess intact microvasculature contiguous with the brain has widespread research and clinical applications.

**NEW & NOTEWORTHY** Measuring the retinal microvasculature at high altitude provides a surrogate for cerebral microvasculature, offering potential insight into consequence of cerebral hypoxia in critical illness. This study demonstrates dynamic regulation of the retina during rapid ascent and prolonged exposure to high altitude and is the first to demonstrate sex-specific differences in retinal microvasculature at high altitude. The ability to dynamically assess intact microvasculature contiguous with the brain has widespread research and clinical applications.

## Graphical abstract

**Figure:**
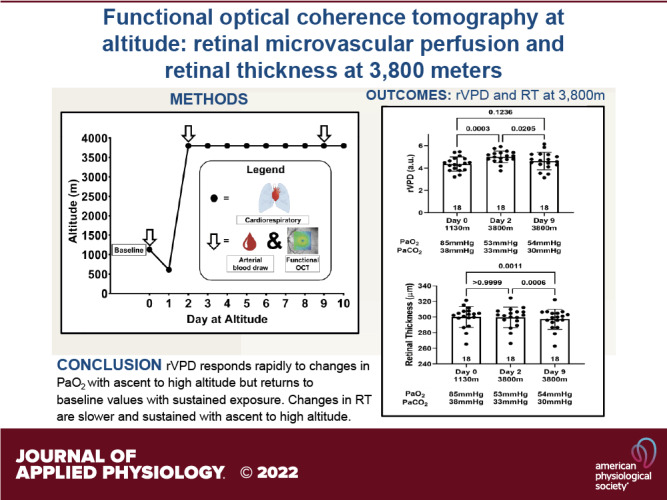

## INTRODUCTION

Tissue hypoxia compromises vital organ function including the brain, and is thus a serious consequence of several cardiorespiratory illnesses including obstructive sleep apnea, chronic obstructive pulmonary disease, pneumonia, pulmonary edema, stroke, and chronic heart failure (1). To protect the brain against tissue hypoxia, the cerebral microvasculature plays a critical role in local blood flow regulation to ensure effective perfusion (2, 3). Our ability to assess the cerebral microvasculature is thus important to understand the effects of hypoxia in cardiovascular and cardiorespiratory diseases that impair oxygen delivery.

Common techniques to measure regional cerebrovascular responses to hypoxia include magnetic resonance imaging (MRI), positron emission tomography (PET), transcranial Doppler (TCD) ultrasound, and near-infrared spectroscopy (NIRS). Unfortunately, MRI and PET are expensive and have limited availability outside the well-resourced hospital setting. Techniques such as TCD and NIRS are more cost-effective and available, however, as with the other techniques, neither provides an assessment of the cerebral microvasculature. A readily available, noninvasive technique capable of providing a proxy for cerebral microvasculature measurements is necessary to advance our understanding of the cerebrovascular consequences of hypoxia associated with cardiorespiratory diseases.

The retina, which lines the back of the eye, includes both microvasculature and neural tissue. As the posterior eye is an outcrop of the brain, the regulation of blood supply to the retina closely resembles that of the brain (4). Both intraretinal and cerebral vascular networks are controlled almost exclusively by myogenic and metabolic mechanisms (4, 5). Thus, measuring the retinal microvasculature likely provides a window into cerebrovascular regulation downstream of large conduit arteries (6). Importantly, noninvasive visualization of these microvascular beds is now possible through modern advances in optical coherence tomography (OCT) (7–9).

OCT provides noninvasive, high-resolution ocular images (7–11) and is the standard of care for assessing retinal conditions in ophthalmic clinics. We recently demonstrated that OCT has sufficient sensitivity to allow for microvascular functional OCT (fOCT), a technique to quantify structural microvascular changes in response to physiological stimuli (12). With each scan, we can obtain quantifiable dynamic properties including retinal vascular perfusion density (rVPD). rVPD is defined as the percentage of a measured area occupied by blood vessels, and thus offers a surrogate assessment of blood supply to the tissue. OCT also provides a sensitive and reproducible measure of retinal thickness (RT), spanning the inner limiting membrane to the retinal pigment epithelium, which reflects changes in vascular and neural tissue volume.

Very little research has focused on sex-specific differences in cerebrovascular ocular blood flow and its regulation often because male and female eyes were traditionally thought to be similar. Although estrogen has been postulated to have protective properties against certain cardiovascular and ocular diseases (13), based on its role to promote vasodilation and increased blood flow (14, 15), testosterone has been shown to have the opposite effect (16). Few studies have investigated sex-specific differences in retinal microvascular blood flow regulation at high altitude, and therefore little is known about the acute and sustained effects of hypobaric hypoxia in males and females.

To shed light on the effects of sustained hypobaric hypoxia on the retinal microvasculature in males and females, we used fOCT to explore dynamic microvascular changes associated with acute and sustained exposure to high altitude ($Pa_{O}{}_{{}_{2}}$: <60 mmHg). Previously, we found an increase in retinal vascular perfusion and thickness during an acute (3-min) bout of hypoxia (12). Based on these findings, we tested the hypothesis that rVPD and RT would increase acutely with hypoxia and that these measures would be directly related to the magnitude of hypoxemia ($Pa_{O}{}_{{}_{2}}$). We also tested the hypothesis that fOCT would be able to detect acute increases, followed by a reduction in rVPD and RT during acute and sustained exposure to hypobaric hypoxia, respectively. Lastly, as a secondary outcome, we aimed to investigate whether rVPD and RT were different between males and females.

## MATERIALS AND METHODS

### Ethics and Participant Recruitment

The study was conducted on members of the White Mountain Research Expedition team. Healthy volunteers (7 females and 11 males) were verbally recruited to participate in a research expedition to investigate cerebral and cardiorespiratory responses following initial ascent to and residence at altitude. The study was conducted at the Barcroft Research Station (3,800 m) in the White Mountains in California, United States, in August 2019. The results herein reflect the ophthalmology study pertaining to this research expedition.

All study participants consulted with their family physicians for a medical clearance before the expedition. However, participants were not screened for eye diseases. All participants were aged 18–60, nonsmokers, and reported no history of cardiopulmonary, neurological, or metabolic disease. Participants were excluded if they had a body mass index (BMI) >35 kg/m^2^, were taking any prescription medication other than female participants taking hormonal birth control, or had previous eye surgery or gross anatomic abnormality. No participants took acetazolamide or corticosteroids (as a prophylactic or treatment for acute mountain sickness) and were asked to abstain from consuming caffeine for at least 12 h before data collection. All study participants were native lowlanders residing permanently at <1,200 m, and no study participants had traveled to high altitude (>3,000 m) in the 12 mo preceding the expedition.

Ethical approval was received in advance through Calgary Conjoint Human Research Ethics Board (REB18-0374), Mount Royal University Human Research Ethics Board (Protocol 101879), the University of British Columbia (H19-01734), and the University of North Texas Health Science Center (Protocol 2019-110). This study adhered to the standards set by the Canadian Tri-Council Policy Statement for research with human participants, consistent with the Declaration of Helsinki, except for registration in a database. All participants provided verbal and written informed consent before voluntarily participating in the study.

### Ascent Profile

**Figure 1.**
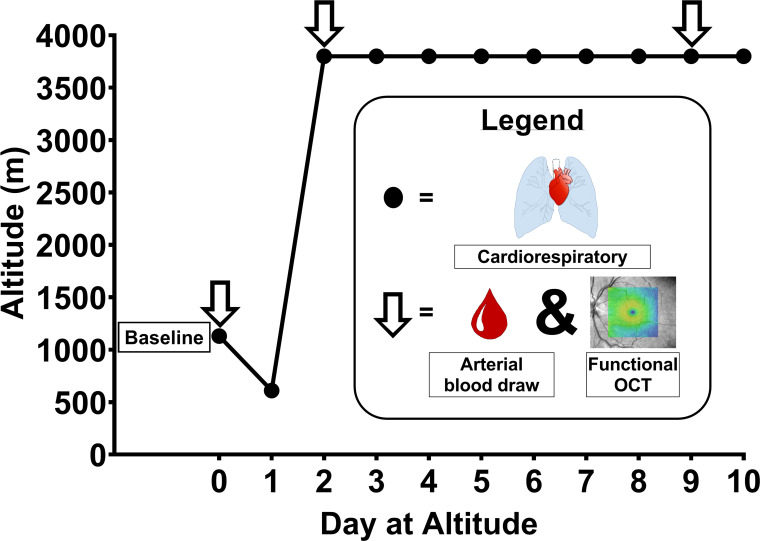
Ascent profile and data collection days. Cardiorespiratory variables (filled circles) were obtained once at low altitude and every morning at high altitude between 0600–0900 local time. Arterial blood (arrow) was obtained once at low altitude (*day 0*) and on *days 2* and *9* of high altitude between 0800–1500 local time. Retinal images (arrow) were taken on *days 0*, *2*, and *9* between 0700–1900 local time. Figure adapted from Bird et al. (17).

Over the course of 1 wk before ascent, baseline measurements (*day 0*) were obtained at low altitude (1,130 m; P_ATM_: ∼665 mmHg) in Calgary, Alberta, Canada. Participants were then flown to Las Vegas, Nevada, United States (610 m) before driving up to the Barcroft Research Station, California, United States (3,800 m; P_ATM_: ∼484 mmHg), and resided at this altitude for 10 days and 10 nights (Fig. 1). Study volunteers were accommodated in the Barcroft Laboratories, which included a standard three-meals a day (western diet) and dorm-style accommodation. All participants were encouraged to drink nonalcoholic beverages throughout the day. While residing at altitude, all participants followed a sleep routine, which included lights out between 2300 and 0700. Retinal images, cardiorespiratory variables, and arterial blood gases were obtained on *day 2* (within 24 h of arrival) and *day 9* to assess the effects of acute and sustained exposure to high altitude, respectively. The atmospheric pressure (P_ATM_) at each altitude was not measured directly, but rather calculated using a standard equation [see Bird et al. (17)], and reported in Table 1 to illustrate the relative reduction in P_ATM_ and partial pressure of inspired oxygen $PI_{O_{2}}$ with ascent.

**Table 1.** Summary of arterial blood parameters and cardiorespiratory on day 0, and in response to acute ascent (day 2) and sustained exposure (day 9) to altitude

	Calgary Lab	Barcroft Lab		
Altitude	1,130 m	3,800 m		
P_ATM_	665 mmHg	484 mmHg		
$PI_{O_{2}}$	130 mmHg	92 mmHg		
	*Day 0*	*Day 2*	*Day 9*	*P* value
Arterial blood				
$Sa_{O_{2}}$, %	96.3 ± 0.96	87.3 ± 3.1*	88.6 ± 1.9	<0.001
$Pa_{CO}{}_{{}_{2}}$, mmHg	37.9 ± 3.7	33.3 ± 3.2*	30.4 ± 1.9*	<0.001
$Pa_{O}{}_{{}_{2}}$, mmHg	84.8 ± 9.1	52.5 ± 5.1*	54.5 ± 4.0*	<0.001
Stimulus index, $Pa_{CO}{}_{{}_{2}}$/$Pa_{O}{}_{{}_{2}}$	0.45 ± 0.1	0.64 ± 0.1*	0.56 ± 0.1*^a^	<0.001
pH	7.41 ± 0.02	7.43 ± 0.2	7.42 ± 0.01	0.08
[HCO_3_^−^], mmol/L	24.2 ± 1.8	22.0 ± 2.2*	19.6 ± 1.4*^a^	<0.001
[Hb], g/L	150.7 ± 13.0	153.2 ± 13.4	165.4 ± 13.0*^a^	<0.001
Hct (PCV %)	43.7 ± 3.4	43.8 ± 2.8	46.6 ± 2.9*^a^	<0.001
O_2_ content, mL/dL	20.1 ± 1.5	18.5 ± 1.6*	20.4 ± 1.6^a^	<0.001
Cardiorespiratory				
HR, beats/min	64.0 ± 12.4	74.7 ± 10.1*	68.7 ± 11.4	0.006
MAP, mmHg	87.7 ± 10.3	87.8 ± 12.7	83.6 ± 9.2	0.07
V̇_E_, L/min	8.9 ± 3.2	10.3 ± 2.8	10.2 ± 1.8	0.08

[Hb], hemoglobin concentration; Hct (PCV %), hematocrit and packed cell volume; [HCO_3_^−^], bicarbonate concentration; HR, heart rate; MAP, mean arterial pressure; $PI_{O_{2}}$, partial pressure of inspired oxygen; V̇_E_, ventilation, *P* value: main effect of time from one-way repeated-measures ANOVA or linear mixed model with Bonferroni post hoc tests if values were missing (missing data included 3 participants who did not have an arterial blood draw). *Values different from *day 0*; ^a^values different from *day 2*.

### Measurements

#### Cardiorespiratory.

Cardiorespiratory variables were obtained once at low altitude and every morning at high altitude between 0600 and 0900 local time with participants in a fasted state. All cardiorespiratory variables were obtained in the supine position following a minimum 3-min baseline with the participant resting quietly with their eyes closed and with white noise headphones to reduce external distraction. For cardiovascular measurements, participants were instrumented with an automated brachial blood pressure monitor (model BP786n; Omron, San Ramon, CA) and a peripheral pulse oximeter (Masimo SET Rad-5, Danderyd, Sweden) placed on the left middle finger for heart rate (HR, beats/min). Three brachial blood pressure measurements were obtained and averaged. Mean arterial pressure (MAP, mmHg) was calculated as 1/3 systolic arterial pressure + 2/3 diastolic arterial pressure. Ventilation (V̇_E_, L/min) was obtained using a respirometer (nSpire Haoscale, Colorado). Participant weight was also recorded daily.

#### Arterial blood.

Arterial blood was obtained once at low altitude (*day 0*) and on *days 2* and *9* of high altitude between 0800 and 1500 local time. While supine, topical sterilization and local anesthesia (EMLA topical cream; 2.5% lidocaine/2.5% prilocaine cream) were applied. Arterial blood samples were drawn from the radial artery using preheparinized, self-filling arterial blood syringes (PICO50, Radiometer). Samples were immediately analyzed using a portable analyzer and cartridges (Abbott iSTAT, CG4+ and CHEM8+ cartridges; Abbott, Mississauga, Ontario, Canada). Arterial blood samples were analyzed for $Pa_{O}{}_{{}_{2}}$ (mmHg), $Sa_{O_{2}}$ (%), $Pa_{\text{C}O}{}_{{}_{2}}$ (mmHg), [HCO_3_^−^] (mM), pH, hematocrit (%), and hemoglobin concentration ([Hb]; g/L). All samples were automatically corrected for body temperature (37°C) and atmospheric pressure conditions. From these measurements, the stimulus index ($Pa_{\text{C}O}{}_{{}_{2}}$/$Pa_{O}{}_{{}_{2}}$) (18, 19), which takes into account the simultaneous but conflicting effects of hypocapnia and hypoxia on brain blood flow, was calculated for each day (18, 19). In addition, arterial oxygen content ($\text{C}a_{O_{2}}$) was calculated as: $\text{C}a_{O_{2}}$$=\left(1.36\times \left[Hb\right]\times \frac{Sa_{O_{2}}}{100}\right)+(0.003\times Pa_{O}{}_{{}_{2}})$, where [Hb] = hemoglobin (g/dL); $Sa_{O_{2}}$ = arterial oxygen saturation (%; or SpO_2_); and $Pa_{O}{}_{{}_{2}}$ = partial pressure of arterial oxygen (mmHg); 1.36 is the binding capacity of oxygen to Hb; and 0.003 is the fraction of free oxygen dissolved in blood (at 37°C).

#### Acute Mountain sickness.

Acute Mountain Sickness (AMS) symptoms were self-reported daily using the 2018 Lake Louise AMS Score (20). AMS was defined as a total score of at least three points, with a headache score of at least one point (20). For each participant, an AMS Clinical Functional Score (CFS) was assigned to assess the effect of AMS symptoms on activities. CFS ranges from 0 (not at all) to 3 (had to be evacuated to a lower altitude). No evacuations were required.

### Optical Coherence Tomography

#### Retinal data acquisition.

Retinal images were collected during seated steady-state rest on *Days 0, 2,* and *9*. Retinal scans were conducted throughout the day, in the same room, between the hours of 0700–1900. For each participant, scans were obtained on average at the same time of day ±4 h. All images were collected using a Cirrus HD 5000 spectral-domain OCT system (OCT, Zeiss optics, Toronto, ON, Canada). The Cirrus HD 5000 OCT [optical source: super luminescent diode (SLD), 840 nm] was used to capture 6 × 6 mm area on the retina with a 2-mm depth to produce a 6 mm × 6 mm × 2 mm scan of the eye (Image resolution: 512 × 128 × 1,024) with an axial and transverse resolution of 5 µm and 15 µm, respectively. To minimize motion artifact while scanning, eye-tracking technology centered on the fovea was used. A minimum of two retinal images were captured during the final 2 min of a 5-min steady-state rest.

#### Retinal data analysis.

All raw retinal images were analyzed by a custom-made automated pipeline written in the Python programming language by M.A.S. This pipeline ensured a standardized approach for denoising and vessel detection. Image-processing steps included retinal layer projection to generate vessels from the three-dimensional (3-D) image, coregistration, and realignment using a rigid body transformation for all images of each participant. This process was followed by denoising, contrast enhancement, and vessel segmentation using Frangi’s algorithm. Projection artifacts were removed using a two-step process. First, the vertical and horizontal band artifacts were removed using the robust principal component analysis that can distinguish vessels from artifacts. Then, the circular-shaped artifacts were removed using Frangi’s vesselness filter to extract only tubular-shaped structures (vessels) in the image. The output from Frangi’s algorithm was then used to determine the probability of each pixel being a blood vessel. A global thresholding approach was applied to make a final binary image. Retinal vascular perfusion density (rVPD), i.e., the percentage of an area occupied by the vasculature, was then calculated as the sum of all pixels belonging to blood vessels divided by the total number of pixels in the image, expressed as arbitrary units (a.u.). The same algorithm parameters were applied to all images from all participants without any human intervention.

To calculate retinal thickness, the inner limiting membrane (ILM) and the retinal pigment epithelium (RPE) 3-D surfaces were extracted using the Iowa Reference Algorithms software (Retinal Image Analysis Lab, Iowa Institute for Biomedical Imaging, Iowa City, IA) (21–23). The distances between these surfaces were measured to generate a two-dimensional thickness map around the macula. This process was also automated, requiring no human intervention. The mean of the highest quality 2–3 images for each individual was used in the rVPD and RT analysis. Images were rejected if the signal strength was <8/10 or if there were prominent artifacts (i.e., movement).

### Statistical Analysis

All continuous data are presented as means ± SD. Data were assessed for normality using the Shapiro–Wilk test. Retinal, cardiorespiratory, and arterial blood data were compared across days using one-way repeated-measures ANOVAs or a linear mixed model if data were missing. The changes in rVPD and RT between days with age and weight as covariates were also determined using an ANCOVA. Male and female data were compared across days using a mixed model. All pairwise comparisons used a Bonferroni correction for multiple comparisons. A repeated-measures correlation was used to investigate the relationship between absolute measures of rVPD and arterial blood gases on *days 0* and *2*. A linear regression was used to investigate the relationship between changes in retinal VPD and thickness and changes in arterial blood gases following acute ascent and sustained exposure to high altitude. All tests were two-tailed, and statistical differences were assumed with a *P* value <0.05. Statistical analyses were performed using SPSS statistical software, Version 25.0, manufactured by IBM Corporation (SPSS Inc., Chicago, IL). Repeated-measure correlations were analyzed using RStudio (v. 1.2) and the rmcorr R package (https://cran.r-project.org/web/packages/rmcorr) (v. 0.4.4) (RStudio, Inc. Boston, MA). Graphical representations were made using Prism GraphPad, Version 9.1, (GraphPad Software, San Diego, CA).

## RESULTS

Eighteen healthy volunteers (7 females and 11 males) participated in this study (age: 29 ± 8 yr; BMI = 24.7 ± 3.6 kg/m^2^). Daily weight measurements revealed that participant weighed less on *day 9* (72.7 ± 13.9 kg) relative to *day 0* (74.3 ± 13.9 kg; *P* < 0.001) and *day 2* (74.3 ± 14.2 kg; *P* = 0.002).

### Arterial Blood and Cardiorespiratory Responses to Acute and Sustained High-Altitude Exposure

Table 1 provides a summary of the arterial blood and cardiorespiratory responses during steady-state rest on *days 0, 2,* and *9*. A more comprehensive analysis and discussion of the ventilatory and renal acid-base changes following acute ascent to and residence at altitude in this cohort have been previously described by Bird et al. (24).

#### Arterial blood gases.

Relative to *day 0*, acute ascent to altitude resulted in arterial hypoxemia ($Pa_{O}{}_{{}_{2}}$: Δ −32.4 ± 6.8 mmHg; $Sa_{O_{2}}$: Δ −9.0 ± 2.7%; *P* < 0.001), and respiratory alkalosis ($Pa_{\text{C}O}{}_{{}_{2}}$: Δ −4.4 ± 1.4 mmHg; *P* < 0.001) on *day 2*, which was partially compensated for by renal bicarbonate excretion (HCO_3_^−^: Δ −2.1 ± 1.4 mmol/L; *P* < 0.001). Subsequently, pH was unchanged on *day 2* (pH: Δ 0.01 ± 0.02; *P* = 0.05). The stimulus index was elevated on *day 2*, a response driven by a large reduction in $Pa_{O}{}_{{}_{2}}$. Arterial oxygen content ($\text{C}a_{O_{2}}$) was also reduced upon acute ascent ($\text{C}a_{O_{2}}$: Δ −1.6 ± 1.3 mL/dL; *P* < 0.001). On *day 9*, $Pa_{O}{}_{{}_{2}}$ and $Sa_{O_{2}}$ remained low compared with *day 0* ($Pa_{O}{}_{{}_{2}}$: Δ −29.7 ± 8.9 mmHg; $Sa_{O_{2}}$: Δ −7.6 ± 1.7%; *P* < 0.001) and were not different from *day 2*, whereas $Pa_{\text{C}O}{}_{{}_{2}}$ was further reduced compared with *day 1* ($Pa_{\text{C}O}{}_{{}_{2}}$: Δ −7.1 ± 2.7 mmHg; *P* < 0.001). Accordingly, the stimulus index decreased relative to *day 2* (*P* < 0.001) but remained elevated compared with *day 0* (*P* < 0.001). HCO_3_^−^ was further reduced (HCO_3_^−^: Δ −4.5 ± 1.5 mmol/L; *P* < 0.001) and pH remained unchanged (pH: Δ 0.001 ± 0.03; *P* > 0.99). As expected, there was no change in hemoglobin and hematocrit on *day 2*, but both were elevated on *day 9* relative to *day 0* (*P* < 0.001) and *day 2* (*P* < 0.001). Subsequently, by *day 9*, $\text{C}a_{O_{2}}$ had returned to low altitude baseline values ($\text{C}a_{O_{2}}$: Δ 0.1 ± 2.2 mL/dL; *P* > 0.99).

#### Cardiorespiratory.

On *day 2*, ventilation was slightly increased (V̇_E_: Δ1.3 ± 2.2 L/min; *P* = 0.08). This was associated with the chronic hypoxic ventilatory response. Heart rate was elevated on *day 2* (HR: Δ10 ± 10 beats/min; *P* = 0.006), but then normalized to *day 0* values by *day 9* (*P* = 0.4). Mean arterial pressure (MAP) was unchanged at high altitude (*P* = 0.07).

### Acute Mountain Sickness

On *day 2*, 12 participants reported Acute Mountain Sickness (AMS) symptoms based on the 2018 Lake Louise AMS Score (Median, IQR: 1.5, 0.25–2.75). Four participants met the clinical definition of AMS. Seven participants reported a score of one on the AMS CFS signifying the presence of symptoms, but not severe enough to change activity or itinerary (20). By *day 9*, only one study participant remained symptomatic, reporting a score of 1 related to mild fatigue/weakness. RT and rVPD were not significantly associated with total AMS scores (*r^2^* = 0.01; *P* = 0.55, *r^2^* = 0.02; *P* = 0.48), AMS CFS (*r^2^* = 0.02, *P* = 0.39; *r^2^* = 0.01; *P* = 0.55), or headache scores (*r^2^* = 0.01, *P* = 0.56; *r^2^* = 0.01; *P* = 0.51).

### Optical Coherence Tomography in Response to Acute and Sustained High-Altitude Exposure

#### Retinal vascular perfusion density.

**Figure 2.**
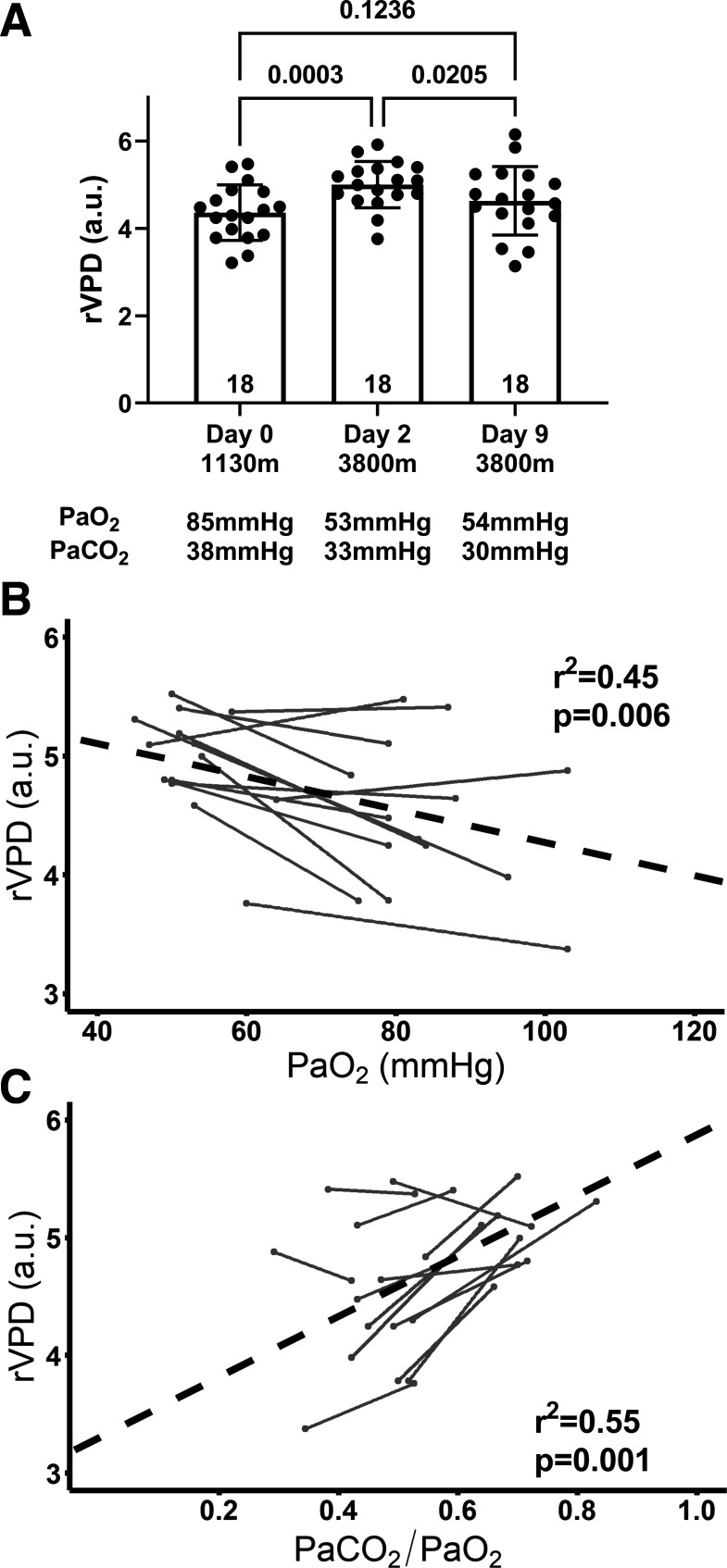
Retinal vascular perfusion density (rVPD) increased significantly on *day 2* following acute ascent (*A*) (*n* = 18). This response was inversely related to $Pa_{O}{}_{{}_{2}}$ (*B*) and directly related to the stimulus index ($Pa_{\text{C}O}{}_{{}_{2}}$/$Pa_{O}{}_{{}_{2}}$) (*n* = 14) (*C*). By *day 9*, rVPD decreased and was no longer different relative to *day 0* (*n* = 13). *A*: one-way repeated-measures ANOVAs with Bonferroni post hoc tests. *B* and *C*: repeated-measures correlation. Individual lines illustrate data from individual subjects, left point day *0* and right point day *2*.

For rVPD, there were main effects for time (*P* < 0.001) and sex (*P* = 0.03), with a significant interaction (*P* = 0.03). rVPD increased on *day 2* (*day 2*: 5.0 ± 0.5 a.u. vs. *day 0*: 4.4 ± 0.6 a.u.; *P* < 0.001; Fig. 2*A*) and was inversely related to $Pa_{O}{}_{{}_{2}}$ (*R^2^* = 0.45; *P* < 0.01; Fig. 2*B*), $\text{C}a_{O_{2}}$ (*R^2^* = 0.31; *P* = 0.02), and directly related to the stimulus index (*R^2^* = 0.55; *P* = 0.001; Fig. 2*C*). By *day 9*, rVPD decreased and was no longer different relative to *day 0* (*day 0*: 4.4 ± 0.6 a.u. vs. *day 9*: 4.6 ± 0.8 a.u.; *P* = 0.12; Fig. 2*A*).

#### Retinal thickness.

**Figure 3.**
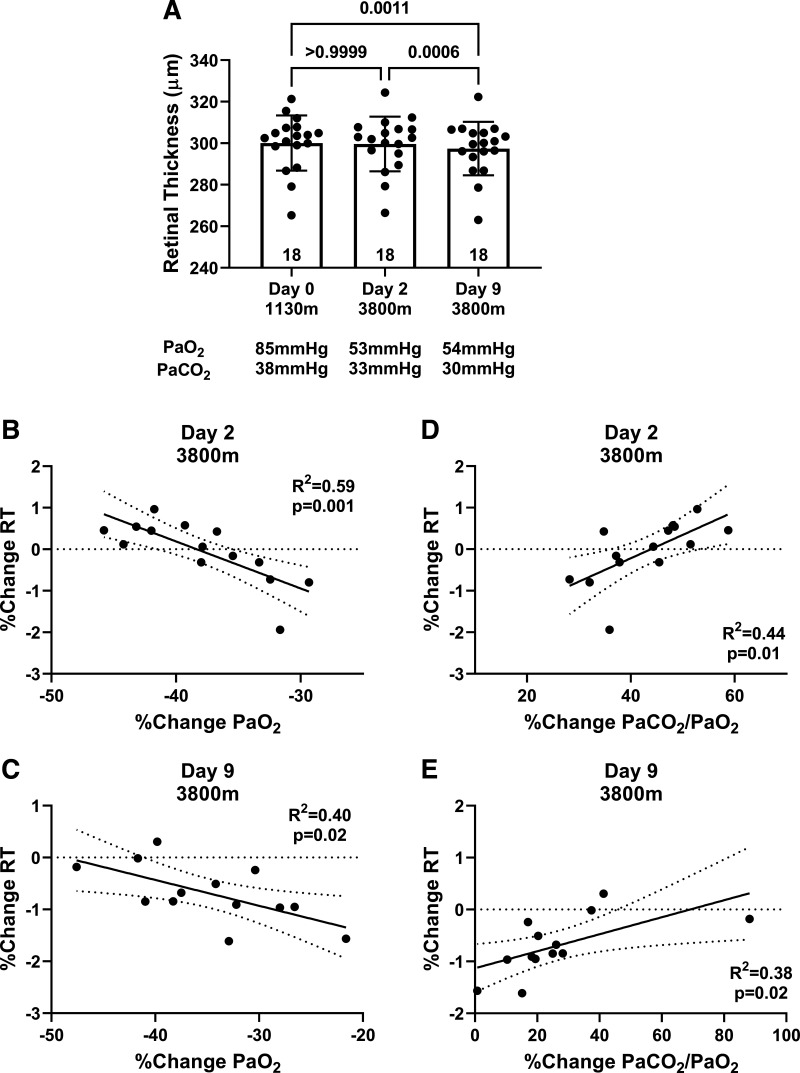
Retinal thickness (RT) was not affected by acute ascent. However, RT was significantly reduced on *day 9* compared with *day 0* and *day 2* (*A*) (*n* = 18). RT changes relative to *day 0* were strongly related to changes in $Pa_{O}{}_{{}_{2}}$ on *day 2* (*B*) (*n* = 14) and *day 9* (*C*) (*n* = 13) and stimulus index on *day 2* (*D*) (*n* = 14) and *day 9* (*E*) (*n* = 13). *A*: one-way repeated-measures ANOVAs with Bonferroni post hoc tests. *B–E*: linear regression.

For RT, there was a main effect of time (*P* < 0.001). RT was not affected by acute ascent (*day 0*: 300.1 ± 13.3 μm vs. *day 2*: 299.6 ± 13.1 μm; *P* > 0.99) but was reduced by *day 9* (*day 9*: 297.4 ± 12.9 μm; *P* < 0.001) relative to *day 0* and *day 2* (Fig. 3*A*). The results of the ANCOVA with weight loss as a covariate showed that the main effect of time on RT remained significant, suggesting weight loss contributed somewhat to the observed changes in RT but did not have a dominant effect. RT changes relative to *day 0* were also related to changes in $Pa_{O}{}_{{}_{2}}$ on *day 2* (*R^2^* = 0.59; *P* = 0.001; Fig. 3*B*) and *day 9* (*R^2^* = 0.40; *P* = 0.02; Fig. 3*C*) and the stimulus index on *day 2* (*R^2^* = 0.44; *P* = 0.01; Fig. 3*D*) and *day 9* (*R^2^* = 0.38; *P* = 0.02; Fig. 3*E*).

### Sex-Related Differences

#### Demographics.

Male (*n* = 11) and female (*n* = 7) participants did not differ in age (M: 29 ± 8, F: 32 ± 9; *P* = 0.77). However, males were taller (M: 177 ± 8 cm, F: 165 ± 9 cm; *P* = 0.01) and heavier (M: 81 ± 13 kg, F: 63 ± 7 kg; *P* = 0.01) than their female counterparts. BMI was not different (M: 25.6 ± 2.7, F: 23.4 ± 4.8; *P* = 0.25).

#### Arterial blood and cardiorespiratory.

Baseline arterial blood values were not different between males and females (Table 2). However, on *day 2*, males had higher [Hb] (*P* = 0.02), Hct (%PCV) (*P* = 0.001), and HCO_3_^−^ (*P* = 0.02), which may have translated to a higher $\text{C}a_{O_{2}}$ (*P* = 0.003) on *day 9* compared with females (Table 2). Cardiorespiratory parameters including V̇_E_ (*P* = 0.11), HR (*P* = 0.76), and MAP (*P* = 0.92) were not different between males and females at baseline or across the expedition (Table 2). Similarly, neither total AMS scores (M: 2.3 ± 2.4, F: 1.7 ± 1.2; *P* = 0.34) nor CFS (M: 0.5 ± 0.5, F: 0.3 ± 0.5; *P* = 0.63) were different.

**Table 2.** Male and female arterial blood and cardiorespiratory values on day 0, day 2, and day 9

Parameter	*Day 0*		*Day 2*		*Day 9*	
	Males (*n* = 9)	Females (*n* = 6)	Males (*n* = 9)	Females (*n* = 6)	Males (*n* = 9)	Females (*n* = 6)
Arterial blood						
$Sa_{O_{2}}$ , %	96.0 ± 1.0	96.7 ± 0.8	86.8 ± 2.8	88.0 ± 3.6	88.4 ± 2.2	88.8 ± 1.7
$Pa_{\text{C}O}{}_{{}_{2}}$, mmHg	39.7 ± 2.2	35.2 ± 4.1	35.0 ± 2.0	30.98 ± 3.0	31.3 ± 0.9	29.4 ± 2.2
$Pa_{O}{}_{{}_{2}}$, mmHg	83.6 ± 9.5	86.7 ± 8.2	51.4 ± 4.7	54.0 ± 5.8	54.4 ± 4.5	54.5 ± 3.7
Stimulus index, $Pa_{CO}{}_{{}_{2}}$/$Pa_{O}{}_{{}_{2}}$	0.5 ± 0.1	0.4 ± 0.1	0.7 ± 0.1	0.6 ± 0.1	0.6 ± 0.1	0.5 ± 0.1
pH	7.4 ± 0.01	7.4 ± 0.03	7.4 ± 0.02	7.4 ± 0.03	7.4 ± 0.01	7.4 ± 0.01
[HCO_3_^−^], mmol/L	25.1 ± 1.5	22.8 ± 1.5	23.2 ± 2.0	**20.4 ± 1.3**	20.1 ± 1.0	18.9 ± 1.5
[Hb] , g/L	155.6 ± 13.1	142.5 ± 8.2	159.2 ± 13.2	**143.2 ± 6.0**	173.6 ± 8.6	**151.7 ± 3.5**
Hct (PCV %)	45.0 ± 3.0	41.3 ± 3.1	45.8 ± 1.8	**41.0 ± 1.7**	48.4 ± 2.1	44.5 ± 2.8
O_2_ content, mL/dL	20.6 ± 1.6	19.2 ± 1.0	18.9 ± 1.8	17.8 ± 0.8	20.86 ± 1.9	**19.0 ± 0.8**
Cardiorespiratory	(*n* = 10)	(*n* = 6)	(*n* = 10)	(*n* = 6)	(*n* = 10)	(*n* = 6)
HR, beats/min	65.3 ± 14.2	62.0 ± 9.7	73.6 ± 10.6	76.5 ± 9.9	68.0 ± 12.4	69.8 ± 10.7
MAP, mmHg	88.5 ± 11.2	86.3 ± 9.5	87.7 ± 11.6	87.8 ± 15.5	84.6 ± 8.7	81.9 ± 10.6
V̇_E_, L/min	9.9 ± 3.6	7.4 ± 1.7	11.1 ± 3.3	9.1 ± 1.3	10.5 ± 1.7	9.5 ± 1.9

[Hb], hemoglobin concentration; Hct (PCV %), hematocrit and packed cell volume; [HCO_3_^−^], bicarbonate concentration; HR, heart rate; MAP, mean arterial pressure; V̇_E_, ventilation. Bold values are significantly different from males: HCO_3_^−^, *P* = 0.02; [Hb] *day 2*, *P* = 0.02; [Hb] *day 9*, *P* < 0.001; Hct, *P* = 0.001; O_2_ content, *P* = 0.003. Missing data included 3 participants who did not have an arterial blood draw (2 M, 1 F) and 2 (1 M, 1 F) with missing cardiorespiratory data. Data were analyzed with a linear mixed model with Bonferroni post hoc tests.

#### Optical coherence tomography.

**Figure 4.**
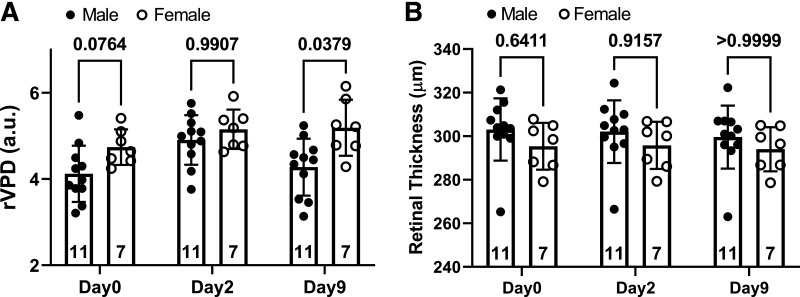
Retinal vascular perfusion density (rVPD) was significantly reduced in males (*n* = 11) compared with females (*n* = 7) on *day 9* (*A*). Retinal thickness (RT) showed no sex-specific differences across the days (*B*). Two-way repeated-measures ANOVA with Bonferroni post hoc tests.

rVPD was not different between males and females on *day 0* (M: 4.1 ± 0.7 a.u., F: 4.7 ± 0.7 a.u.; *P* = 0.08) or *day 2* (M: 4.9 ± 0.6 a.u., F: 5.2 ± 0.5 a.u.; *P* = 0.99). By *day 9*, however, rVPD was lower in males (4.3 ± 0.7 a.u.) than in females (5.2 ± 0.9 a.u.; *P* = 0.04) (Fig. 4*A*). Among male participants, there was a significant effect of time on rVPD. rVPD was increased on *day 2* relative to *day 0* (*P* < 0.001) and *day 9* (*P* = 0.001). There were no differences among female participants across days (*P* = 0.11). RT did not show evidence of sex-related differences on *day 0* (M: 303.1 ± 14.3, F: 295.4 ± 10.1; *P* = 0.64), *day 2*, (M: 302.1 ± 14.4, F: 295.8 ± 10.1; *P* = 0.92), or *day 9* (M: 299.6 ± 14.4, F: 294.0 ± 9.4; *P* > 0.99) (Fig. 4*B*).

## DISCUSSION

The aim of the current study was to test the hypothesis that rVPD and RT would increase acutely with hypoxia and that these measures would be directly related to the magnitude of $Pa_{O}{}_{{}_{2}}$. We also tested the hypothesis that fOCT would be able to detect acute increases, followed by a reduction in rVPD and RT during acute and sustained exposure to hypobaric hypoxia, respectively. As a secondary outcome, we aimed to investigate whether rVPD and RT were different between males and females. The results of the current study suggest *1*) differential time course and metabolic regulation of retinal microvasculature compared with retinal thickness at altitude, *2*) that fOCT provides a novel technique to detect absolute changes in the retinal microvasculature in response to acute and sustained hypobaric hypoxia, and *3*) sex-specific differences in rVPD following sustained exposure to high altitude.

### Retinal Vascular Perfusion Density

The retina is metabolically active and, like the brain, has a high demand for oxygen (25). To support this demand, blood supply to the retina is regulated by two distinct circulations. The inner retinal layers, comprising ganglion, horizontal, bipolar, and amacrine cells, depend on retinal perfusion from the central retinal artery. The outer retinal layers primarily include the photoreceptors and are supplied by choroid perfusion (26). The results of the current study support arterial hypoxemia as an important local metabolic factor influencing changes in retinal microvascular perfusion. In the present study, rVPD increased upon initial ascent to high altitude, a response that was related to reduced $Pa_{O}{}_{{}_{2}}$ and a larger stimulus index. Following sustained exposure to high altitude, rVPD returned to baseline values, which is consistent with previous reports examining the cerebral (27, 28) and retinal (29) circulation at altitude. For example, using magnified fundus photography and blue-field simulation (BFS), Bosch et al. (29) showed that, in humans, arterial vessel diameter of the temporal superior branch of the retinal artery and blood flow velocity were increased with the initial ascent to high altitude. However, both fundus photography and BFS have limitations related to repeated measures, which will be discussed below (see *Comparison of fOCT to Other Techniques*). Our results are also consistent with other studies of retinal physiology during hypoxia using OCT-angiography (30–33). In a group of young healthy volunteers, Hommer et al. (31) reported a significant increase in perfusion density within the superficial vascular plexus of the retina during hypoxic challenges. Similarly, Sousa et al. (33) reported a significant increase in both the superficial and deep vascular plexuses. In the study, we did not assess rVPD in different retinal layers.

The magnitude of within-subject changes seen with ascent in our study are comparable to other between-subject OCT-angiography studies involving clinical populations (32, 34, 35). For example, in patients with diabetic retinopathy, Agemy et al. (34) found that, compared with healthy volunteers, patients with diabetic retinopathy had a 7% and 13% reduction in capillary perfusion density in the superficial and deep retinal plexuses, respectively. Similarly, Nelis et al. (35) found that patients with cerebral autosomal dominant arteriopathy with subcortical infarcts and leukoencephalopathy had a 6% reduction in deep retinal plexus vessel density. Compared with these clinical studies, the current study was conducted in relatively young and healthy volunteers; follow-up studies will be required to determine how ascent and/or hypoxia affect the retinal circulation of other populations.

### Retinal Thickness

In contrast to our hypothesis, RT did not change upon acute ascent, but rather decreased following sustained exposure to high altitude. Like rVPD, these changes were inversely related to $Pa_{O}{}_{{}_{2}}$ and directly related to the stimulus index. These data support the role of arterial hypoxemia as a factor affecting RT. By *day 9*, nearly all individuals had a reduction in RT compared with *day 0* and *day 2*. However, because $Pa_{O}{}_{{}_{2}}$ was relatively maintained between *day 2* and *day 9*, these data would suggest other factors, beyond arterial hypoxemia, may also be influencing RT. One explanation influencing reduced RT could be related to dehydration, which has been reported with acute and prolonged exposure to high altitude (36). Alternatively, weight loss may have also been the result of time spent at high altitude rather than by changes in diet or increased physical activity. Nonetheless, the use of a weight-loss covariate (a surrogate measure of dehydration) did not abolish the observed significance. Therefore, the decreased thickness of the retina could suggest a selective, possibly neuronal-specific tissue dehydration related to high-altitude physiological adaptation.

Changes in RT were shown to be directly related to choroid thickness and perfusion (37). Therefore, another possible explanation for the observed results may be related to changes in choroid perfusion to the outer retinal layers. The outer retinal layers are an avascular network, and as such, their supply of nutrients and oxygen is dependent on choroid perfusion (4, 38). During hypoxic conditions, choroid vascular perfusion has been shown to be significantly reduced (12). Accordingly, reductions in RT may be due to vascular perfusion insufficiency related to reductions in choroidal perfusion and/or choroid thinning. Further exploration of choroid thickness and choroid vascular perfusion is required to help address these questions.

Regarding the reliability and potential clinical significance of the current findings, a recent literature review of 27 articles looking at the reliability of retinal layer thickness estimates showed that the Cirrus HD-OCT has high intraobserver reliability with intraclass correlation values (>0.9) and a coefficient of repeatability <2 μm (39). Moreover, a recent study estimated that thickness loss in the ganglion cell layer and the combined ganglion cell layer + inner plexiform layer in patients with multiple sclerosis was estimated at 0.5 μm/yr (40). Other clinical studies have shown within-group retinal thickness changes and/or differences between healthy controls as little as 5 µm (41–43). Lastly, in patients with a de novo hypertensive disorder related to pregnancy, compared with baseline (i.e., nonpregnant state), retinal thickness was reduced by 4.35 µm and 2.99 µm at 20 wk and 20–40 wk, respectively (44). Together, these studies suggest that although the observed RT changes in the current study are not necessarily pathological, they do demonstrate the ability of fOCT to detect subtle retinal thickness changes under different physiological conditions that may have important clinical implications within patient cohorts.

### Sex-Related Differences

Despite no significant differences on *day 0* and *day 2*, our data show that rVPD was lower in males following 9 days at altitude than in females. As these differences do not appear until *day 9*, the mechanisms involved in retinal vascular regulation may be, in part, related to sex-specific differences in physiological adaptation to high altitude. HR, MAP, and V̇_E_ were not different between males and females with ascent and short-term residence at high altitude. Therefore, it is less likely that these cardiorespiratory characteristics contributed to the observed differences. On the other hand, we found that although [Hb] and $\text{C}a_{O_{2}}$ were not different between males and females at baseline, by *day 2* [Hb] was higher in males, and remained higher on *day 9*, which corresponded with a higher $\text{C}a_{O_{2}}$ on *day 9*. Increased production of [Hb] is one of the slower (i.e., 1–2 wk) physiological mechanisms involved in acclimatization (45, 46), suggesting the difference seen on *day 2* was likely the result of males having slightly higher baseline values compared with females (*P* = 0.08). On *day 9*, however, males had much higher levels of [Hb], which in turn increases the oxygen-carrying capacity of the blood and facilitates adequate tissue oxygenation. This translated to a higher $\text{C}a_{O_{2}}$ in males than in females, which may explain why rVPD returned to baseline values. These results are similar to those of Iwase et al. (47), who found lower [Hb] in females than in males, which was negatively correlated with ocular blood flow, which the authors attributed to lower blood O_2_ capacity.

Other considerations include the known influence of sex hormones on circulatory control as well as acid-base balance in systemic circulation (48–50). Therefore, we cannot rule out the possibility that sex hormones played a role in our findings and had an influence on the regulation of retinal blood flow. Pertinent to our findings, Faria et al. (51) found that estrogen decreases vascular resistance in the central retinal artery in postmenopausal women, compared with placebo. Similarly, Toker et al. (16) found a positive correlation between serum estrogen levels and ocular blood flow, whereas testosterone had the opposite effect. Given the duration of the expedition (9 days), it is likely that sex hormone concentrations changed throughout, which may have contributed to estrogen-mediated vasodilation. Understanding sex-specific differences in retinal vascular perfusion are important as differences in ophthalmic disease prevalence and incidence among males and females exist (13), suggesting sex hormones play an important role. Unfortunately, the literature on sex-specific differences in retinal blood flow regulation and ocular diseases is sparse, and as such, more work is warranted. In contrast to rVPD, RT showed no sex-specific differences at baseline or in response to acute and sustained hypobaric hypoxia at high altitude. The lack of sex-related differences at baseline corresponds with previous reports of ophthalmic indices at rest using fOCT (12).

### Comparison of fOCT to Other Techniques

The microvasculature is central to the maintenance of tissue health and function, and measurements of retinal circulation provide an opportunity to evaluate an intact microvascular network. Our findings support the use of fOCT to detect absolute dynamic microvascular changes in response to acute and sustained hypobaric hypoxia. As an outcrop of the brain, visualization and quantification of the retinal microvasculature and retinal thickness, as offered with fOCT, provides a novel technique to assess changes in this unique microvascular bed that is contiguous with the brain.

Changes in cerebral and microvascular blood flow in response to stress can be estimated using several different techniques (52). For the past two decades, TCD ultrasound has been the primary means to measure bulk cerebral blood flow at altitude. However, TCD ultrasound measures blood velocity in large conduit arteries, which has two main drawbacks: *1*) Because TCD measures velocity, and not cerebral blood flow, absolute repeated values are affected by factors such as vessel diameter, the quality of the insonation window, and angle of insonation and *2*) blood flow within large conduit arteries does not necessarily represent changes in blood flow within the microvasculature. The latter can be overcome by measuring changes within the retinal microvasculature, which has been performed at altitude using fundus photography with micrometric methods (53, 54), BFS (29), and Laser-Doppler flowmetry (LDF) (29). These techniques, however, are not without their own limitations.

Both fundus photography with micrometric methods and BFS use subjective approaches to estimate vessel diameter and flow, respectively, and are therefore less reliable for repeated measures (55–57), including looking at prolonged exposure to hypoxic stress over several days. In addition, repeatability with fundus photography is largely dependent on operator experience and as such demonstrates significant interobserver differences (55). These subjective measures affect reliability and make these techniques impractical for clinical use (55, 57). In the present study, all raw images were analyzed using a custom-made automated pipeline to ensure a standardized approach without human intervention, subsequently reducing operator bias.

LDF measures a Doppler shift caused by the flow of red blood cells to determine blood flow at the optic nerve head (55). However, LDF has limited small vessel resolution (6) and tissue depth (58), issues with data interpretation across studies (58, 59), and variable reproducibility (6, 60). OCT, on the other hand, has become the standard for structural ocular imaging due to its ability to directly obtain noninvasive images with a spatial resolution of 4 μm and scanning depth of 500 μm (11). Furthermore, OCT has good within- and between-day reproducibility (55), and unlike TCD ultrasound, retinal images can be acquired using consistent dimensions (6 mm × 6 mm) and location within the macula, which allows for direct comparison of absolute measures and responses.

Overall, our findings demonstrate that fOCT can detect absolute dynamic changes in rVPD and RT. Importantly, the measurable changes using this approach mirror that of cerebral blood velocity (27, 28) at altitude, lending support to the idea that blood supply to the retina is closely related to that of the brain (4, 5), offering a potential window into cerebrovascular regulation. As the retinal microvasculature provides a surrogate for the cerebral microvasculature, measuring changes within the intact retinal microvascular beds provide an opportunity to study physiological mechanisms associated with acute and sustained exposure to hypobaric hypoxia, which may provide insight into cerebral hypoxia in critical illness.

### Limitations

Functional OCT is still evolving, and therefore the results of the current study should be interpreted along with the following limitations. *1*) Changes in blood flow and blood volume to tissue occur if there is a metabolic change within the tissue. In the current study, we do not know the metabolic consumption of O_2_ in the retina, nor do we know if this changes with sustained hypobaric hypoxia/high altitude. Estimates of O_2_ uptake depend on blood flow and the arteriovenous O_2_ difference in accordance with the Fick principle. Having only surrogate measures of total blood supply, we cannot make assumptions regarding O_2_ uptake or the underlying changes in neural activity. *2*) Sex hormones, including estrogen, progesterone, and testosterone, were not measured. Similarly, as some female participants were on hormonal birth control, this may increase intersubject variability. In addition, we did not record the phase of menstrual cycle in female participants. Given the known phasic fluctuations in estrogen and progesterone levels, these could have influenced rVPD throughout the expedition. *3*) Neither RT nor rVPD was related to AMS scores. These findings may be due to limitations associated with subjective, self-reported scales. *4*) The current study had a relatively small sample size (*n* = 18) dictated by expedition logistics, which could affect the reliability of the findings. Despite the small sample relative to laboratory studies, our sample is comparable to previously published high-altitude studies of similar nature (27, 61–63). *5*) Retinal scans were not conducted at the same time of day, nor were participants fasted for the scans; however, there were no significant differences between blood glucose levels (*P* = 0.18) between days. Nonetheless, the effect of diurnal changes on systemic and ocular variables may have introduced a potential source of error in our measurements. *6*) Participants were not formally prescreened for eye diseases.

### Conclusion

Overall, these data suggest differential time course and regulation of the retina microvasculature compared with retinal thickness at high altitude. In the current study, we show that rVPD responds rapidly to changes in $Pa_{O}{}_{{}_{2}}$, but returns to baseline values with sustained exposure, a response similar to bulk cerebral blood flow. In contrast, changes in retinal thickness were slower and sustained with altitude. These data also support the significance of $Pa_{O}{}_{{}_{2}}$ on retinal microvasculature and thickness but suggest additional factors, such as tissue dehydration and choroid perfusion and/or thinning, may affect retinal thickness. The current study demonstrates sex-specific differences in retinal vascular perfusion density at high altitude. Finally, the current study supports the use of fOCT to detect absolute changes in tissue dynamics, including retinal vascular perfusion density and retinal thickness. The ability of fOCT to assess an intact microvascular bed that is contiguous with the brain will provide novel insights with widespread research and clinical applications.

## GRANTS

This work was funded by a University of Calgary URGC Grant (to R.J.A.W.), Natural Sciences and Engineering Research Council of Canada (NSERC) Discovery grants (to T.A.D.: RGPIN-2016–04915; to R.J.A.W.: RGPIN-2015–03941). Additional financial support was provided by the Alberta Government Student Temporary Employment Program and NSERC Undergraduate Student Research Assistantships. Salary support was obtained from the Canadian Institutes of Health Research (CIHR), Libin Cardiovascular Institute Post-Doctoral Fellowship in Women’s Cardiovascular Health and the NSERC Brain CREATE Program (to J.B.), CIHR Post-Doctoral Fellowship (to A.V.I.), Alberta Innovates Graduate Scholarship, NSERC Brain CREATE, T. Chen Fong Scholarship in Medical Imaging Science and T. Chen Fong Research Excellence Scholarship (to M.A.S.), the Parker B. Francis Post-Doctoral Fellowship and NSERC BRAIN CREATE Program (to N.G.J.), and CIHR (to S.R.R.). American Heart Association Grant-in-Aid was provided to C.A.R. (17GRNT33671110).

## DISCLOSURES

N.G.J. and R.J.A.W. have submitted a patent application for the use of optical coherence tomography as a method to interrogate cardiorespiratory function. G.E.F. is a Michael Smith Foundation for Health Research Scholar and R.J.A.W. is an Alberta Innovates Senior Scholar. None of the other authors has any conflicts of interest, financial or otherwise, to disclose. 

## AUTHOR CONTRIBUTIONS

N.G.J., T.A.D., and R.J.A.W. conceived and designed research; N.G.J., J.D.B., T.A.D., C.A.R., and R.J.A.W. performed experiments; J.B., M.A.S., A.V.I., and U.A. analyzed data; J.B., A.V.I., T.A.D., and R.J.A.W. interpreted results of experiments; J.B. and M.A.S. prepared figures; J.B. drafted manuscript; J.B., M.A.S., A.V.I., N.G.J., G.E.F., J.D.B., S.R.R., T.A.D., C.A.R., N.Z-D., and R.J.A.W. edited and revised manuscript; J.B., M.A.S., A.V.I., N.G.J., G.E.F., J.D.B., S.R.R., T.A.D., C.A.R., N.Z-D., U.A., and R.J.A.W. approved final version of manuscript.
